# Use of ^18^F-fluoride PET to determine the appropriate tissue sampling region for improved sensitivity of tissue examinations in cases of suspected periprosthetic infection after total hip arthroplasty

**DOI:** 10.3109/17453674.2011.594232

**Published:** 2011-09-02

**Authors:** Hyonmin Choe, Yutaka Inaba, Naomi Kobayashi, Hiroyuki Ike, Chie Aoki, Kazuya Shizukuishi, Naoyuki Iwamoto, Yohei Yukizawa, Takashi Ishida, Tomio Inoue, Tomoyuki Saito

**Affiliations:** ^1^Department of Orthopaedic Surgery; ^2^Department of Radiology, School of Medicine, Yokohama City University, Yokohama, Japan; Correspondence: yute@yhc.att.ne.jp

## Abstract

**Background and purpose:**

The accurate diagnosis of periprosthetic infection requires assessment of intraoperative tissues. These must be sampled from the appropriate sites.

We used ^18^F-fluoride positron emission tomography (PET) to identify sites of inflammation in order to improve the sensitivity of histopathology, microbiological culture, and real-time PCR in total hip arthroplasty (THA) patients.

**Patients and methods:**

23 THA patients (23 hips) scheduled for revision surgery (the revision group) and 17 uninfected THA patients (23 hips; control group) were enrolled. Uptake was classified into major, minor, and no uptake. To evaluate the association between the ^18^F-fluoride uptake and intraoperative tissue results in the revision group, we calculated their sensitivity on each of the major, minor, and no-uptake sides.

**Results:**

17 revision patients showed major uptake and all were diagnosed as having septic loosening from intraoperative tissue results. Minor uptake was observed in the other 6 revision patients and all were diagnosed as having aseptic loosening. Apart from 3 cases that showed minor uptake regions, control subjects showed no uptake. In the revision group, the sensitivities of histopathology, microbiological culture, real-time PCR separately and also in combination were 0.78, 0.58, 0.96, and 0.96, respectively, on the major ^18^F-fluoride uptake sides, 0.0, 0.0, 0.1, and 0.1 on the minor-uptake sides, and 0, 0, 0.18, and 0.18 on the no-uptake sides.

**Interpretation:**

Our findings suggest that preoperative assessment of major uptake of ^18^F-fluoride markedly improves the accuracy of tissue sampling, and thus the sensitivity of subsequent tissue examinations. More definitive diagnosis of periprosthetic infection is therefore possible.

Due to absence of physical inflammation and negative culture results, it may be difficult to differentiate low-grade prosthetic infections from aseptic loosening (Kobayashi et al. 2008, Trampuz and Zimmerli 2008, Moojen et al. 2010). To allow accurate diagnosis in such cases, molecular methods such as polymerase chain reaction (PCR)-based assays and histopathological examination should be combined (Bauer et al. 2006). Thus, determination of the correct sampling region—in broad terms, whether it is on the acetabular side or the femoral side in total hip arthroplasty (THA) patients—is important.

The aim of the present study was not to differentiate septic from aseptic loosening using ^18^F-fluoride positron emission tomography (PET), but to determine whether the results of tissue examinations in THA patients are affected by the sampling location, classified as major, minor, or no-uptake sides in terms of ^18^F-fluoride uptake.

## Patients and methods

This prospective study was approved by the institutional review board of Yokohama City University Hospital (01-08-2006, No 204). A consecutive series of 41 THA patients experiencing pain (43 hips) and 17 THA patients with no complications (23 hips) who gave informed consent were investigated with ^18^F-fluoride PET between 2006 and 2010. 23 of the 41 THA patients experiencing pain (23 of 43 hips), with a mean age of 73 (56–89) years, who were scheduled to undergo revision surgery were classified as the revision group and were enrolled in the study. To determine the normal range of ^18^F-fluoride uptake values, 17 pain-free THA patients (23 hips) with a mean age of 68 (32–74) years and with no evidence of loosening from radiographic, serological, or physical examination were enrolled as the control group. The mean time after primary surgery was 14 (2–28) years in the revision group and 7 (2–20) years in the control group. The control group was followed for mean 2.2 (1–3) years after fluoride PET scanning by regular physical, radiographic, and serological examinations.

**Figure 1.**
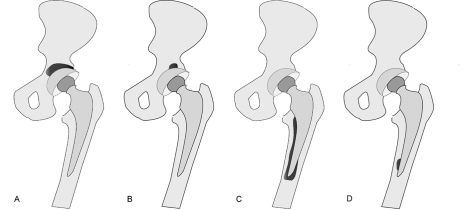
Definitions used for the ^18^F-fluoride PET uptake patterns on the acetabular and femoral sides. A. Major-uptake region on the acetabular side: ^18^F-fluoride uptake has spread to more than 50% of the acetabular component with an SUV_max_ of > 5. B. Minor-uptake region on the acetabular side: ^18^F-fluoride uptake has localized in less than 50% of the acetabular component with an SUV_max_ of > 5. C. Major-uptake region on the femoral side: ^18^F-fluoride uptake has spread to more than 50% of the femoral component with an SUV_max_ of > 5. D. Minor-uptake region on the femoral side: ^18^F-fluoride uptake has localized in less than 50% of the femoral component with an SUV_max_ of > 5.

The pattern of ^18^F-fluoride PET uptake was classified into 3 categories: major uptake, minor uptake, and no uptake, using the following definitions (Figure 1). An SUV_max_ of > 5 covering more than 50% of the implant was defined as major uptake. An SUV_max_ of >5 covering less than 50% of the implant was defined as minor uptake. When the SUV_max_ was less than 5, this was defined as no uptake. The same criteria were applied to the acetabular side and the femoral side.

### PET scanning

PET images were obtained using a SET 2400 W machine (Shimadzu, Kyoto, Japan) equipped with 20.0-cm and 59.5-cm transverse fields of view and producing 63 image planes with a 3.125-mm interval. The transverse resolution at the center of view was 4.2 mm, and the full width-half maximum was 5.0 mm. A whole-body image was obtained 40 min after the injection of 185 MBq ^18^F-fluoride in 10 mL of 0.9% saline solution using the multiple-bed position technique. 4 to 5 sections from the head to the thigh were imaged. Attenuation-corrected transverse images were reconstructed using the ordered-subsets expectation maximization algorithm into 128 × 128 matrices with pixel dimensions of 4.0 mm in-plane and 3.125 mm axially. Coronal images with a 9.8-mm section thickness were also reconstructed from attenuation-corrected transverse images for visual interpretation (Tayama et al. 2007).

The maximal standardized uptake value (SUV_max_) was measured axially on the acetabular and femoral sides of each joint by 2 investigators, and the higher of the measurements was used. We then compared these values in the revision group and the control group. Statistical analysis was performed using the unpaired t-test and a p-value of less than 0.01 was considered to be significant.

### Diagnosis of infection in the current study

A definitive diagnosis of infection was made from the evaluation of intraoperative specimens obtained from both the acetabular and femoral sides in all patients in the revision group. These tissue samples were evaluated by histopathological examination, microbiological culture, and real-time PCR for the detection of bacterial DNA. When at least 1 of these 3 examinations showed a positive finding, a definitive diagnosis of periprosthetic infection was made. To obtain consistency between the ^18^F-fluoride uptake and intraoperative results, the sensitivity of these intraoperative results on the ^18^F-fluoride major-uptake, minor-uptake, and no-uptake sides were calculated separately.

### Histopathological examination

Frozen sections were used to establish an rapid diagnosis intraoperatively, and permanent sections were used for diagnosis postoperatively. Infiltration of 10 or more neutrophils per high-power field (400× magnification, with a field diameter of 0.6 mm) was defined as acute inflammation, and was considered to be suggestive of infection (Lonner et al. 1996).

### Microbiological culture

All specimens were processed using standard microbiological culture (aerobic and anaerobic), and bacteria were allowed to grow for up to 7 days using Nissui Tube Gifu anaerobic medium (GAM) (Nissui Pharmaceutical, Tokyo, Japan), which was semi-solid. A VITEK 2 compact device (bioMérieux, Inc., Durham, NC) was used for automated identification of microorganisms in accordance with the manufacturer's instructions.

### Real-time PCR

Bacterial DNA was extracted from the intraoperative tissues using a Qiagen DNA mini kit (Qiagen, Valencia, CA) and then analyzed by quantitative real-time PCR using the Light Cycler 2.0 system (Roche Diagnostics GmbH, Mannheim, Germany). 2 different primer and probe sets were used: methicillin-resistant staphylococcus (MRS) PCR using a methicillin-resistant Staphylococcus aureus (MRSA) detection kit (Roche Diagnostics GmbH, Mannheim, Germany) and broad-range universal PCR, as previously described (Kobayashi et al. 2006, 2009). The same program settings were used in both cases: an initial hot start at 95°C for 10 min, followed by 45 cycles at 95°C for 10 seconds, 55°C for 10 seconds, and 72°C for 12 seconds.

## Results

Major-uptake regions were found in 17 patients in the revision group—on the acetabular side or the femoral side, or both—and a definitive infection was confirmed in each of these 17 patients. The 6 remaining patients in the revision group showed minor-uptake regions on one or both sides, and no infections were diagnosed in these patients (Table 1).

**Table 1.** Results of PET, histopathological examination, microbiological culture, and real-time PCR in the revision group

			^18^F–fluoride PET		Postoperative diagnosis from intraoperative specimens			
A	B	C	D	E	F	G	H	I
1	75	F	5.1 / 6.7	Major / Major	+ / –	+ / +	+ / +	*Staphylococcus hominis–hominis* (MRS)
2	73	F	5.1 / 3.1	Major / –	+ / –	– / –	+ / +	Negative
3	65	M	9.1 / 2.5	Major / –	+ / –	+ / –	+ / –	MRSE
4	73	F	8.6 / 4.9	Major / –	+ / –	+ / –	+ / +	MRSE
5	65	M	5.6 / 13	Minor / Major	– / N/A	– / –	– / +	Negative
6	63	F	7.7 / 10	Minor / Major	– / +	– / –	+ / +	*Streptococcus agalactiae*
7	75	M	11 / 12	Major / Major	+ / +	+ / +	+ / +	*Staphylococcus auricularis* (MRS)
8	77	F	17 / 3.5	Major / –	+ / –	– / –	+ / –	Negative
9	65	M	6.8 / 9.8	Minor / Major	– / –	– / –	– / +	Negative
10	71	F	10 / 11	Major / Major	– / +	– / –	– / +	Negative
11	70	M	12 / 10	Major / Major	+ / +	+ / +	+ / +	Non–hemolytic streptococcus
12	56	F	9.8 / 6.1	Major / Minor	+ / –	– / –	N/A / N/A	Negative
13	89	M	5.8 / 6.8	Major / Major	+ / +	+ / +	+ / +	*Streptococcus sanguinis*
14	70	F	14 / 2.3	Major / –	– / –	+ / –	+ / –	*Staphylococcus warneri*
15	82	F	9.0/13	Major / Major	– / +	+ / –	+ / +	Aerobic Gram–positive bacillus
16	83	F	15 / 11	Major / Minor	+ / –	– / –	+ / –	Negative
17	69	F	6.8 / 14	Major / Major	+ / +	+ / +	+ / +	MRSE
18	78	M	5.2 / 2.3	Minor / –	– / –	– / –	– / –	Negative
19	69	F	6.8 / 2.3	Minor / –	– / –	– / –	– / –	Negative
20	59	M	2.8 / 9.0	– / Minor	– / –	– / –	– / –	Negative
21	83	M	1.7 / 6.2	– / Minor	– / –	– / –	– / –	Negative
22	83	F	9.9 / 4.5	Minor / –	– / –	– / –	– / –	Negative
23	73	M	7.4 / 3.3	Minor / –	– / –	– / –	– / –	Negative

N/A: not available. A Case B Age C Sex D SUV _max_ (acetabular/femoral) E Uptake pattern (acetabular/femoral) F Histopathological examination (acetabular/femoral) G Microbiological culture (acetabular/femoral) H Real-time PCR (acetabular/femoral) I Bacterial strain

In the control group, minor-uptake regions were observed in 3 patients but no major-uptake regions were found in any of these patients. In addition, none of the control patients showed any evidence of infection or loosening in radiographic, serological, or physical examinations during the follow-up period.

The mean SUV_max_ of the septic loosening patients was 11 (5–17) and that of the 6 aseptic loosening patients was 7 (5–10). The mean SUV_max_ of the control group was 4 (3–6). The overall differences between the septic loosening patients and the control group, and also between the aseptic loosening patients and the control group in terms of the SUV_max_ were statistically significant (p < 0.001 and p < 0.01, respectively).

**Figure 2.**
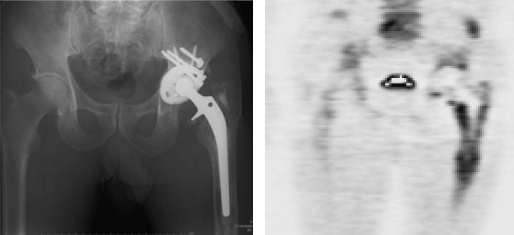
A. Patient no. 5 (revision group) with a radiolucent line around the femoral implant. B. ^18^F-fluoride PET image showing minor uptake with an SUV_max_ of 5.6 on the acetabular side and major uptake with an SUV_max_ of 13 on the femoral side. In this patient, real-time PCR was positive only for tissues sampled from the femoral side, suggesting the existence of localized infection around the femoral component.

**Figure 3.**
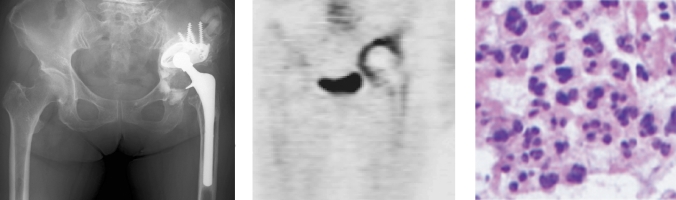
A. Patient no. 8 (revision group) with severe loosening of the cup side. B. ^18^F-fluoride PET image. Massive ^18^F-fluoride uptake on the acetabular side suggested that the focal point of inflammation was localized around the acetabular component. The SUV_max_ was 17 on the acetabular side (white arrow) and 3.5 on the femoral side, indicating major uptake only on the acetabular side. C. Histopathological examination indicated infection with a minimum of 5 HPF (×400) containing 10 or more neutrophils. In this patient, the histopathological examination and real-time PCR results were both positive only for tissues sampled from the acetabular side, suggesting the existence of localized infection on the membrane around the acetabular component.

**Figure 4.**
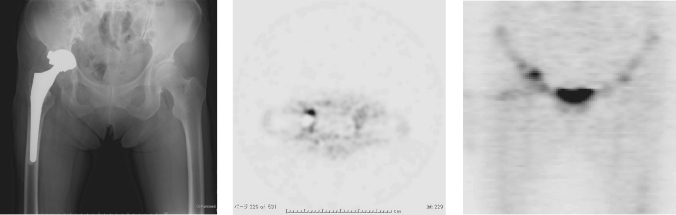
A. Patient no. 22 (revision group) with a horizontally-moved acetabular implant. B. ^18^F-fluoride PET axial image showing a minor-uptake region with an SUV_max_ of 9.9 on the acetabular side. C. ^18^F-fluoride PET coronal image showing a minor-uptake region on the acetabular side and a no-uptake region with an SUV_max_ of 4.5 on the femoral side. This patient was diagnosed as having aseptic loosening, as no positive results were obtained in any of the tissue examinations

Major-uptake regions were found on 24 sides in 17 patients (14 sides on the acetabular side and 10 sides on the femoral side). Definitive infection was found in 23 of the 24 sides with major uptake. The sensitivity of the 3 methods for diagnosis of infection was calculated separately for major-, minor-, and no-uptake sides in the revision group: 0.78, 0.58, 0.96, respectively, and in combination (0.96). In contrast, the corresponding sensitivities of these tests for the minor-uptake sides were 0.00, 0.00, 0.10, and 0.10, and for the no-uptake sides they were 0.00, 0.00, 0.18, and 0.18, respectively (Table 2). Figures 2 and 3 show representative ^18^F-fluoride images in septic loosening cases and Figure 4 shows representative ^18^F-fluoride images in aseptic loosening cases

**Table 2.** The sensitivity of histopathological examination, microbiological culture, and real-time PCR—individually and in combination—in analyzing the ^18^F-fluoride major-, minor- and no-uptake sides in the revision group

	On major-uptake side (24 sides)	On minor-uptake side (11 sides)	On no-uptake side (11 sides)
Histopathological examination			
Positive	18	0	0
Negative	5	11	11
Side not available	1	–	–
Sensitivity	0.78	0	0
Microbiological culture			
Positive	14	0	0
Negative	10	11	11
Sensitivity	0.58	0	0
Real-time PCR			
Positive	22	1	2
Negative	1	9	9
Side not available	1	1	–
Sensitivity	0.96	0.1	0.18
Combined			
Positive	23	1	2
Negative	1	10	9
Sensitivity	0.96	0.1	0.18

## Discussion

A robust diagnosis of periprosthetic infection requires that a combination of testing methods should be undertaken (Trampuz and Zimmerli 2008). It must be noted that the accuracy of all diagnostic tests is limited by the appropriateness of the tissue sampling. In other words, if intraoperative tissue specimens are collected from an uninfected site, false-negative results will be obtained. We found that sampling of intraoperative tissues to establish a definitive diagnosis of infection after THA should be from the ^18^F-fluoride major-uptake side to give reliable results.

^99m^Tc-labeled bone scintigraphy, which is regarded as having a dynamic capacity similar to that of ^18^F-fluoride to provide bone remodeling data, is also a useful test for the diagnosis of periprosthetic complications of the hip (Gallo et al. 2004). Using a time-difference radioisotope uptake technique, a triple-phase bone scanning protocol can be useful for diagnosing periprosthetic infection with a sensitivity of 88% and a specificity of 90% (Nagoya et al. 2008). ^18^F-fluorodeoxy glucose (FDG)-PET is one of the best known of these imaging technologies, and it has been applied extensively to the diagnosis of periprosthetic infections of the hip (Zhuang et al. 2001, Reinartz et al. 2005, Delank et al. 2006, Pill et al. 2006, Stumpe and Strobel 2006, Chryssikos et al. 2008). Chryssikos et al. (2008) have emphasized that optimal diagnostic criteria can differentiate septic loosening from aseptic loosening at a hip prosthesis with 85% sensitivity and a specificity of 93%. However, the reliability of this in discriminating between aseptic and septic inflammation remains controversial (Chacko et al. 2002, Stumpe et al. 2004, Delank et al. 2006).

^18^F-fluoride is a well-established positron-emitting bone-seeking agent, and its uptake reflects both blood flow and remodeling of bone. With the recent improvements in imaging efficiency, ^18^F-fluoride PET has been recognized for its usefulness in diagnosing the regional characterization of skeletal disorders such as malignant and benign skeletal legio lesion, bone metastasis, or Paget's disease (Installe et al. 2005, Grant et al. 2008). However, there have been no reports to date of the use of ^18^F-fluoride PET imaging in cases of periprosthetic infection. Although our patient series was not large, our findings strongly suggest that major uptake of ^18^F-fluoride periprosthetically in THA cases is of value in determining the correct tissue sampling side and in giving the possibility of preserving the implant on another side. However, it is still unclear whether the use of ^18^F-fluoride improves the sensitivity and specificity regarding differentiation of septic loosening from aseptic loosening, and more clinical cases will have to be evaluated in future in order to confirm its usefulness.

One limitation of the present study is the small number of cases of aseptic loosening in the patient cohort. We were therefore unable to calculate the sensitivity and specificity of ^18^F-fluoride PET for the diagnosis of septic loosening as described above. However, ^18^F-fluoride PET was found to be diagnostically useful in cases of periprosthetic infection, with no false positives arising in our aseptic loosening cases or in the control group, and no false negatives observed in the cases of septic loosening.

## References

[CIT0001] Bauer TW, Parvizi J, Kobayashi N, Krebs V (2006). Diagnosis of periprosthetic infection. J Bone Joint Surg (Am).

[CIT0002] Chacko TK, Zhuang H, Stevenson K, Moussavian B, Alavi A (2002). The importance of the location of fluorodeoxyglucose uptake in periprosthetic infection in painful hip prostheses. Nucl Med Commun.

[CIT0003] Chryssikos T, Parvizi J, Ghanem E, Newberg A, Zhuang H, Alavi A (2008). FDG-PET imaging can diagnose periprosthetic infection of the hip. Clin Orthop.

[CIT0004] Delank KS, Schmidt M, Michael JW, Dietlein M, Schicha H, Eysel P (2006). The implications of 18F-FDG PET for the diagnosis of endoprosthetic loosening and infection in hip and knee arthroplasty: results from a prospective, blinded study. BMC Musculoskelet Disord.

[CIT0005] Gallo J, Kaminek M, Myslivecek M, Zapletalova J, Spicka J (2004). Validity of bone scintigraphy for the diagnosis of periprosthetic complications in hydroxyapatite-coated total hip arthroplasty. Acta Chir Orthop Traumatol Cech.

[CIT0006] Grant FD, Fahey FH, Packard AB, Davis RT, Alavi A, Treves ST (2008). Skeletal PET with 18F-fluoride: applying new technology to an old tracer. J Nucl Med.

[CIT0007] Installe J, Nzeusseu A, Bol A, Depresseux G, Devogelaer JP (2005). Lonneux M. (18)F-fluoride PET for monitoring therapeutic response in Paget's disease of bone. J Nucl Med.

[CIT0008] Kobayashi N, Bauer TW, Tuohy MJ, Lieberman IH, Krebs V, Togawa D, Fujishiro T, Procop GW (2006). The comparison of pyrosequencing molecular Gram stain, culture, and conventional Gram stain for diagnosing orthopaedic infections. J Orthop Res.

[CIT0009] Kobayashi N, Procop GW, Krebs V, Kobayashi H, Bauer TW (2008). Molecular identification of bacteria from aseptically loose implants. Clin Orthop.

[CIT0010] Kobayashi N, Inaba Y, Choe H, Iwamoto N, Ishida T, Yukizawa Y, Aoki C, Ike H, Saito T (2009). Rapid and sensitive detection of methicillin-resistant Staphylococcus periprosthetic infections using real-time polymerase chain reaction. Diagn Microbiol Infect Dis.

[CIT0011] Lonner JH, Desai P, Dicesare PE, Steiner G, Zuckerman JD (1996). The reliability of analysis of intraoperative frozen sections for identifying active infection during revision hip or knee arthroplasty. J Bone Joint Surg (Am).

[CIT0012] Moojen DJ, van Hellemondt G, Vogely HC, Burger BJ, Walenkamp GH, Tulp NJ, Schreurs BW, de Meulemeester FR, Schot CS, van de Pol I, Fujishiro T, Schouls LM, Bauer TW, Dhert WJ (2010). Incidence of low-grade infection in aseptic loosening of total hip arthroplasty. Acta Orthop.

[CIT0013] Nagoya S, Kaya M, Sasaki M, Tateda K, Yamashita T (2008). Diagnosis of peri-prosthetic infection at the hip using triple-phase bone scintigraphy. J Bone Joint Surg (Br).

[CIT0014] Pill SG, Parvizi J, Tang PH, Garino JP, Nelson C, Zhuang H, Alavi A (2006). Comparison of fluorodeoxyglucose positron emission tomography and (111)indium-white blood cell imaging in the diagnosis of periprosthetic infection of the hip. J Arthroplasty (Suppl 2).

[CIT0015] Reinartz P, Mumme T, Hermanns B, Cremerius U, Wirtz DC, Schaefer WM, Niethard FU, Buell U (2005). Radionuclide imaging of the painful hip arthroplasty: positron-emission tomography versus triple-phase bone scanning. J Bone Joint Surg (Br).

[CIT0016] Stumpe KD, Strobel K (2006). 18F FDG-PET imaging in musculoskeletal infection. Q J Nucl Med Mol Imaging.

[CIT0017] Stumpe KD, Notzli HP, Zanetti M, Kamel EM, Hany TF, Gorres GW, von Schulthess GK, Hodler J (2004). FDG PET for differentiation of infection and aseptic loosening in total hip replacements: comparison with conventional radiography and three-phase bone scintigraphy. Radiology.

[CIT0018] Tayama Y, Takahashi N, Oka T, Takahashi A, Aratake M, Saitou T, Inoue T (2007). Clinical evaluation of the effect of attenuation correction technique on 18F-fluoride PET images. Ann Nucl Med.

[CIT0019] Trampuz A, Zimmerli W (2008). Diagnosis and treatment of implant-associated septic arthritis and osteomyelitis. Curr Infect Dis Rep.

[CIT0020] Zhuang H, Duarte PS, Pourdehnad M, Maes A, Van Acker F, Shnier D, Garino JP, Fitzgerald RH, Alavi A (2001). The promising role of 18F-FDG PET in detecting infected lower limb prosthesis implants. J Nucl Med.

